# Commentary: Case Report: Abdominal Lymph Node Metastases of Parathyroid Carcinoma: Diagnostic Workup, Molecular Diagnosis, and Clinical Management

**DOI:** 10.3389/fendo.2021.700806

**Published:** 2021-06-18

**Authors:** Giuseppe Fanciulli, Sergio Di Molfetta, Andrea Dotto, Tullio Florio, Tiziana Feola, Annamaria Colao, Antongiulio Faggiano, NIKE Group, Manuela Albertelli

**Affiliations:** ^1^ NET Unit, Department of Medical, Surgical and Experimental Sciences, University of Sassari—Endocrinology Unit, Sassari, Italy; ^2^ Section of Internal Medicine, Endocrinology, Andrology and Metabolic Diseases, Department of Emergency and Organ Transplantation, University of Bari Aldo Moro, Bari, Italy; ^3^ Endocrinology Unit, IRCCS Ospedale Policlinico San Martino, Genova, Italy; ^4^ Department of Internal Medicine, University of Genova, Genova, Italy; ^5^ IRCCS Ospedale Policlinico San Martino, Genova, Italy; ^6^ Department of Experimental Medicine, “Sapienza” University of Rome, Rome, Italy; ^7^ Neuroendocrinology, Neuromed Institute, IRCCS, Pozzilli, Italy; ^8^ Endocrinology Unit, Department of Clinical Medicine and Surgery, University Federico II, Naples, Italy; ^9^ Endocrinology Unit, Department of Clinical and Molecular Medicine, Sant’Andrea Hospital, Sapienza University of Rome, Rome, Italy

**Keywords:** parathyroid carcinoma, immune checkpoint inhibitors, ipilimumab, nivolumab, pembrolizumab

## Introduction

In the issue of March 2021, Lenschow et al. reported the case of a 46-year-old woman with recurrent, programmed death-ligand-1 (PD-L1) negative, tumor mutational burden (TMB)-high parathyroid carcinoma (PC), who showed stable disease as her best response on imaging, and a three-fold drop in PTH after treatment with intravenous pembrolizumab (1).

Parathyroid carcinoma is a rare neuroendocrine tumour, accounting for <1% of all cases of primary hyperparathyroidism (2). While surgery represents the mainstay of treatment for both the primary tumour and metastasis, patients no longer amenable to surgical resection often receive unsatisfactory systemic therapies including cinacalcet, adjuvant radiotherapy, and alkylating agents (3).

In recent years, modulation of immune checkpoint proteins expression has been accounted as a prominent mechanism for tumour immune evasion and survival, thus paving the way for new therapeutic approaches (4). Of note, monoclonal antibodies targeting the programmed cell death-1 (PD-1)/PD-L1 and/or the cytotoxic T lymphocyte antigen-4 (CTLA-4)-B7 pathway, hereinafter collectively referred as immune checkpoints inhibitors (ICIs), have shown both clinical effectiveness and a favorable safety profile in patients with advanced solid tumours, and have been included in the treatment repertoire of several malignancies (5).

Given the remarkable results obtained by Lenschow et al. with the anti-PD-1 agent pembrolizumab in the above-mentioned case, we performed an extensive search for possible further relevant data sources, including a) full published articles in international online databases (PubMed, Web of Science, Scopus, and Embase); b) preliminary reports in selected international meeting abstract repositories (American Society of Clinical Oncology, ASCO; European Neuroendocrine Tumor Society, ENET; European Society for Medical Oncology, ESMO); c) registered clinical trials in the U.S. National Institutes of Health registry of clinical trials (http://clinicaltrials.gov) and in any primary register of the WHO International Clinical Trials Registry Platform (ICTRP).

## Findings

- The search for full-published articles revealed 263 papers, two of which were pertinent to our aim (one of these being the article by Lenschow et al.). In 2020, Park et al. (6) reported the case of a 65-year-old man with recurrent hyperparathyroidism and histologically confirmed metastatic PC, who showed objective response as defined by the Response Evaluation Criteria in Solid Tumors version 1.1 (RECIST v1.1) (7) after pembrolizumab administration. The tumour was assessed as PD-L1 negative by immunohistochemistry. Mutations in the MSH2 and MSH6 DNA mismatch repair genes, possibly resulting in high replication error at microsatellite loci, were found in tumour samples through comprehensive gene profiling analysis. Therefore, the patient was deemed eligible for treatment with pembrolizumab. Immune blockade of PD-1 resulted in sustained reduction of pulmonary metastatic tumour burden, with concurrent normalization of both calcium and parathyroid hormone levels.
- We found no preliminary reports in the above-mentioned international meeting abstract repositories.
- The search in clinical trial registers revealed two active trials, one of which fully matched our aim. NCT02834013 (DART: Dual Anti-CTLA-4 and Anti-PD-1 Blockade in Rare Tumors) is a Phase 2 study evaluating the effects of nivolumab plus ipilimumab (arm I) versus nivolumab alone (arm II) in patients with rare solid tumours (94 listed histotypes, including PC). The primary outcome is the RECIST v1.1 objective response-rate. Major secondary outcomes include incidence of adverse events, best response, clinical benefit rate, overall survival, and progression free survival. The present study status is “Recruiting.” However, according to a very recent update of the protocol, accrual of parathyroid gland tumours has been closed.

## Discussion

To date, very limited evidence is available about the efficacy of ICIs in patients with PC. With this regard, some points should be taken into account.

PD-L1 expression in pre-treatment tumour samples has been proposed as a marker for clinical response to anti-PD-1/PD-L1 immunotherapy in patients with advanced malignancies, including melanoma, non-small cell lung cancer, kidney cancer, colorectal cancer, and castration-resistant prostate cancer (8, 9). Notably, immunohistochemistry-assessed PD-L1 expression was found in 4/18 patients (22.2%) with histologically confirmed PC (10), thus suggesting that immune checkpoint blockade may have a rationale in the treatment of this type of tumours. While PD-L1–overexpressing tumours tend to have more intense responses, experience with melanoma suggests that PD-1/PD-L1 blockade may be beneficial also in patients with low PD-L1 expression (11–13), therefore a negative PD-L1 status assessment should not definitively preclude the use of ICIs.

There is growing evidence that the TMB can also predict response to ICIs, with the high TMB-patients exhibiting a higher response rate to anti-PD-1/PD-L1 agents possibly due to increased neo-antigen load and T cell infiltration in the tumour microenvironment (14, 15). In a cohort of 16 patients with PC, Kang et al. have recently found three cases with high (>20 m/Mb) TMB through comprehensive genomic profiling (16). Given the higher response rate observed in the high TMB-patients, assessment of mismatch repair status and/or exome sequencing in tumour samples may help identify those patients possibly benefiting the most from administration of anti-PD-1/PD-L1 agents, in this way enabling a more personalized approach to treatment. The above-mentioned cases of PD-L1 negative, TMB-high tumours benefiting from pembrolizumab therapy further support this approach.

Moreover, PD-1 and CTLA-4 are acknowledged to exert non-redundant immunosuppressive effects (17). As there is robust evidence supporting a greater efficacy of the combined PD-1/CTLA-4 blockade over the two monotherapies in advanced solid cancer (18), the possible inclusion of patients with PC in the NCT02834013 trial is giving rise to great expectations. Of note, ICI two-drug combination therapy is under evaluation also in patients with other aggressive endocrine tumours (19–22).

As a further reason of interest, hypocalcemia due to immune-related hypoparathyroidism has been reported as a rare complication following anti-PD-1 therapy initiation in patients with non-parathyroid tumours (23, 24). As a result, mitigation of hypercalcemia could be hypothesized as a beneficial adjunctive effect of anti-PD-1 agents in patients with PC, irrespective of their imaging response assessment.

In summary, currently available treatments for patients with recurrent PC are insufficient. ICIs, which are considered a milestone in oncology, may provide hope for the future therapy of this rare cancer.

## Author Contributions

GF, SDM, AD, TFl, and TFe were responsible for the design, the methodology, the draft preparation, the reviewing, and editing. AC and AF were responsible for the supervision. All authors contributed to the article and approved the submitted version.

## Funding

This work was supported by the Italian Ministry of Education, University and Research (MIUR): PRIN 2017Z3N3YC, and by University of Sassari, Italy: Research Funding 2019-2020 to GF.

## Conflict of Interest

The authors declare that the research was conducted in the absence of any commercial or financial relationships that could be construed as a potential conflict of interest.

The reviewer MG declared a past co-authorship with several of the authors TFe, AC, and AF to the handling editor.
